# Deep Learning Based Detection Tool for Impacted Mandibular Third Molar Teeth

**DOI:** 10.3390/diagnostics12040942

**Published:** 2022-04-09

**Authors:** Mahmut Emin Celik

**Affiliations:** Department of Electrical Electronics Engineering, Faculty of Engineering, Gazi University, Eti mah. Yukselis sk. No: 5 Maltepe, Ankara 06570, Turkey; mahmutemincelik@gazi.edu.tr

**Keywords:** impacted, tooth, detection, deep learning, panoramic radiograph, machine learning, dentistry

## Abstract

Third molar impacted teeth are a common issue with all ages, possibly causing tooth decay, root resorption, and pain. This study was aimed at developing a computer-assisted detection system based on deep convolutional neural networks for the detection of third molar impacted teeth using different architectures and to evaluate the potential usefulness and accuracy of the proposed solutions on panoramic radiographs. A total of 440 panoramic radiographs from 300 patients were randomly divided. As a two-stage technique, Faster RCNN with ResNet50, AlexNet, and VGG16 as a backbone and one-stage technique YOLOv3 were used. The Faster-RCNN, as a detector, yielded a mAP@0.5 rate of 0.91 with ResNet50 backbone while VGG16 and AlexNet showed slightly lower performances: 0.87 and 0.86, respectively. The other detector, YOLO v3, provided the highest detection efficacy with a mAP@0.5 of 0.96. Recall and precision were 0.93 and 0.88, respectively, which supported its high performance. Considering the findings from different architectures, it was seen that the proposed one-stage detector YOLOv3 had excellent performance for impacted mandibular third molar tooth detection on panoramic radiographs. Promising results showed that diagnostic tools based on state-ofthe-art deep learning models were reliable and robust for clinical decision-making.

## 1. Introduction

Dental clinics frequently use different types of radiography with distinct properties. They visualize different regions of interest for diagnosis and further treatment planning [1]. Panoramic radiographs were initially one of the most common visualization techniques in dentistry that scans a wide area with a significantly lower radiation dose [2]. They enable a variety of anomalies, conditions, and lesions to be diagnosed by experts [1,2,3]. However, complex anatomical structures, pathologies, and imaging distortions can make detecting a case or interpreting a critical condition difficult. Computer-assisted diagnostic systems can help clinicians in decision-making [4]. Recently, the introduction of artificial intelligence-based approaches has efficiently overcome the limitations of traditional methods. Automatically identifying the optimal representations, learning features from raw data are used instead of hand-crafted features [5].

Artificial intelligence (AI) refers to systems and devices designed to address real-life problems as creative as human beings treat them by mimicking natural human intelligence and behavior [6,7,8]. Machine learning (ML) is a subset of AI and consists of algorithms to learn from a large set of data that enables computers to learn how to solve a problem by performing a specific task [9,10]. They improve as they experience more data at the task [11]. Deep learning (DL) is a subset of ML and consists of algorithms inspired by structural and functional properties of the human brain, called artificial neural networks [12,13]. They train themselves to learn to perform specific tasks. More extensive neural networks and training them with more data scales the performance up for real-life tasks such as classification, object detection, segmentation, and object recognition [14,15,16]. Convolutional neural networks (CNNs) are a version of the neural networks that include convolution operations in at least one of the layers.

In recent years, DL models have been intensively applied to many fields, including healthcare, which covers a wide range of applications related to medical diagnosis purposes. There has been a growing interest in artificial intelligence-based systems in dentistry. The use of deep learning in dentistry, including orthodontics, periodontology, endodontics, dental radiology, and forensic medicine, has shown promising results in classification, segmentation, and detection tasks [17,18]. Applications are based on teeth, oral structures, pathologies, cephalometric landmarks, bone loss, periodontal inflammation, and root morphology [19,20,21,22,23,24,25,26,27]. According to the number of published research, a smaller number of several initiatives in dental care develop software and digital dental approaches to diagnostic tools [28]. Some of them are CranioCatch, Digital Smile Design (DSD), 3Shape software (3Shape Design Studio and 3Shape Implant Studio), Exocad, and Bellus 3D.

Object detection task refers to determining the coordinates of a specific object in the input data, while classification refers to automatically assigning the objects into pre-determined categories. When the artificial intelligence-based computer-aided systems are engaged in the field, they can:

support clinicians and physicians who are busy all day to avoid misdiagnosis;
help populations with a shortage of radiologists or screening modalities;
help radiologists manage their workloads in large hospitals;
create reports about pathologic or anatomical conditions in panoramic radiographs, which results in saving time;
provide a focus on the education of observers and new graduates in clinics.

Partially or entirely impacted third molars are the most common developmental conditions affecting humans and require surgical intervention [29,30,31]. Tooth position, adjacent tooth, alveolar bone, and surrounding mucosal soft tissue usually cause failed eruption [32]. They may cause pain, tooth decay, swelling, and root resorption for various reasons, while they might asymptomatically indicate other pathologies, like caries, periodontal diseases, cysts, or tumors, around the second or third molar [32,33,34]. Removal of the third molar for severe cases alleviates symptoms and helps the patients’ oral health [32,35,36]. It is one of the most common surgical procedures performed in secondary care in the UK [37].

This work aimed to develop a decision support system that will help dentists. It presents a state-of-the-art artificial intelligence-based detection solution, including deep learning algorithms with multiple convolutional neural networks to mandibular third molar impacted teeth problems in panoramic radiographs. Two different detectors were used, namely Faster RCNN and YOLOv3. While the YOLOv3 was a single-step technique, the Faster RCNN was a two-stage method in which different backbones were needed to finalize the detection process. Three different backbones, ResNet50, AlexNet, and VGG16, were combined with Faster RCNN. The detection performance was evaluated by mean average precision (mAP), recall, and precision. Classification accuracy was also calculated for each model.

### Related Works

It is intended to explore studies directly related to a detection task for third molars on panoramic radiographs.

Faure et al. (2021) proposed an approach to automatically diagnosing impacted teeth with 530 panoramic radiographs. They implemented only one model using Faster-RCNN with ResNet101, identifying impacted teeth with performances between 51.7% and 88.9% [38]. Kuwada et al. (2020) used deep learning models on panoramic radiographs to detect and classify the presence of impacted supernumerary teeth in the anterior maxillary area [4]. It was reported that DetectNet showed the highest accuracy value of 0.96. Zhang et al. (2018) predicted postoperative facial swelling following impacted mandibular third molars extraction using 15 factors related to patients [39]. Orhan et al. (2021) performed a segmentation task to detect third molar teeth using Cone-Beam Computed Tomography [40]. One hundred twelve teeth are used. A precision value of 0.77 was reported. Basaran et al. (2021) developed a diagnostic charting for ten dental situations, including impacted teeth [41]. Faster R-CNN Inception v2 was implemented using 1084 panoramic radiographs. The precision value for the impacted tooth was 0.779.

Other studies performed detection tasks that aimed to recognize automatically and number teeth [19,20,21,42]. Panoramic, periapical, and dental bitewing radiographs were used. Their results were presented for all teeth together instead of a separate analysis for impacted third molars. Moreover, previous works did not include categorization for angulation concerning adjacent teeth.

## 2. Materials and Methods

The Ethical Review Board approved this study at Ankara Yildirim Beyazit University (approval number 2021-69). It was performed following the ethical standards of the Helsinki Declaration.

Panoramic radiographs of 300 patients older than 18 with at least one impacted tooth in the third-molar region were randomly selected from the image database from January 2018 and January 2020. Panoramic radiographs (PRs) with complete or incomplete impacted tooths with complete root formations for patients older than 18 years old were included, while PRs with artifacts, movement, and position-based distortions and incomplete root formations were excluded.

**Figure 1:**
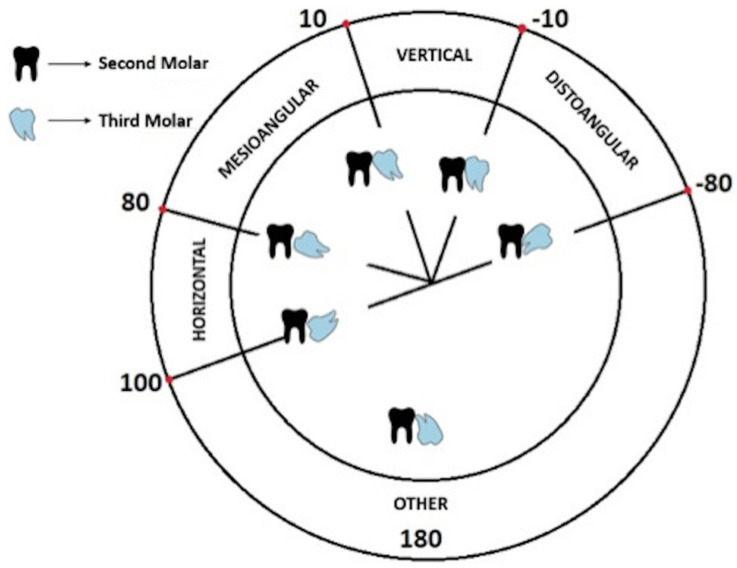
Winter’s mandibular third molar teeth classification scheme [34].

The Winter classification approach was used to categorize mandibular third molar teeth into mesioangular, horizontal positions for both sides [43,44]. The idea was based on the angle between the long axes of the third molar and second molar tooth. While the mesioangular position indicated an angle from 11° to 79°, the horizontal position referred angle from 80° to 100°, as shown schematically in Figure 1.

The original files had DCM format with a resolution of 2943 × 1435. They first converted to PNG using MATLAB, then resized to 640 × 640 before passing to the models. The PyTorch library was used for developing the models. The dataset was analyzed and labeled by an oral and maxillofacial radiologist with more than five years of experience in the field using labelImg [45]. Rectangular bounding boxes enclosing the crown and root of the interested tooth were used. Experiments were performed using k-fold cross-validation (k = 5) instead of a single standard split. It ensures that models were tested on all kinds of potentially tricky cases. The best performance of each fold is determined.

**Figure 2:**
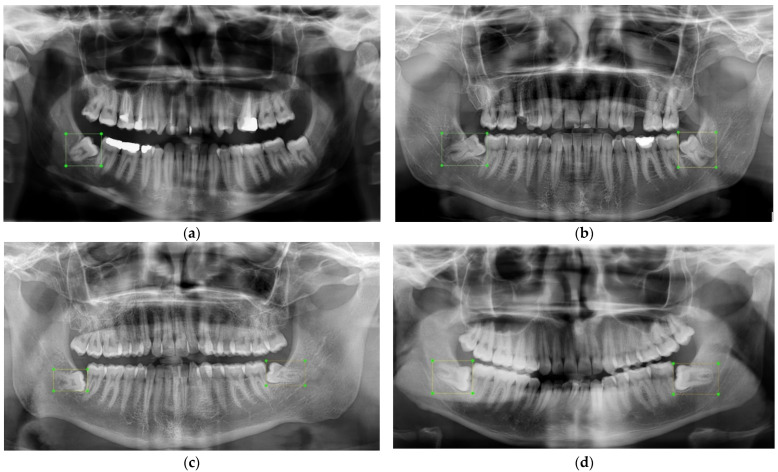
Examples of panoramic radiographs with bounding boxes. (**a**) One impacted tooth—me-sioangular left, (**b**) two impacted teeth—mesioangular left and right, (**c**,**d**) horizontal left and right.

Previous studies on the prevalence of impacted third molars showed that they were twice more likely to be seen in the mandible than in the maxilla [46,47,48,49,50,51,52,53,54]. Almost half of the angulation of incidences was mesioangular. There was no statistically significant difference between the right and left sides. In the light of the preceding findings and the available dataset, mandibular third molars with four classes were chosen to be analyzed. Four classes were defining mandibular third molar teeth that t1, t4, t5, and t8 indicated mesioangular left, horizontal left, mesioangular right and horizontal right impacted teeth respectively. Class distributions were balanced. The total number of impacted third molars was 588 from 440 panoramic radiographs. To keep the dataset balanced for experiments, the number of each class for t1, t4, t5, and t8 was determined as 155, 134, 169, and 130 respectively in the design phase of the work. Figure 2 demonstrates four panoramic radiographs with impacted teeth used as inputs for the proposed detection solution. Figure 2a has only a mesioangular tooth on the left, class t1. Figure 2b has two mesioangular teeth on both sides, classes of t1 and t5. Figure 2c,d has two horizontal teeth on both sides, classes t4 and t8.

To date, the state-of-the-art object detectors are categorized into two classes, namely two-stage methods and one-stage methods [55,56]. Two-stage detectors have proposal-driven mechanisms that first candidate object locations, bounding boxes, are firstly proposed, and then each candidate location is assigned to classes using a convolutional neural network [57]. In contrast, one-stage detectors, with the advantage of being simpler, makes use of anchor boxes to localize and restrict the region and the shape of an object to be detected in the image; in other words, they find bounding boxes in a single step without using region proposals [58,59,60].

The AI-based model development phase includes two detectors, namely Faster RCNN and YOLOv3 [57,58]. YOLOv3 performs the detection in a single-phase, although Faster RCNN is a two-stage technique that needs a backbone as a feature extractor. So, ResNet50, AlexNet, and VGG16 are also used as a backbone and Faster RCNN one at a time.

AlexNet consists of 5 convolutional and three fully connected layers. It features Rectified Linear Units to model a neuron’s output, and provides training on multiple GPUs and overlapping pooling, making the process faster [61]. VGG16 is a convolutional neural network model with 13 convolutional and five pooling layers. Large kernel filters used in AlexNet are replaced with 3 × 3 kernel-sized filters in VGG16 architecture for better performance with ease of implementation [62]. After AlexNet and VGG16, architectures begin to become deeper; however, it makes the back-propagated gradient extremely small sometimes, resulting in saturated or decreased performance. Residual Networks, ResNet50, solves this issue by suggesting identity shortcut connections that skip one or more layers and perform identity mappings [63]. It has a depth of up to 152 layers and reduces the number of parameters needed for a deep network.

**Figure 3:**
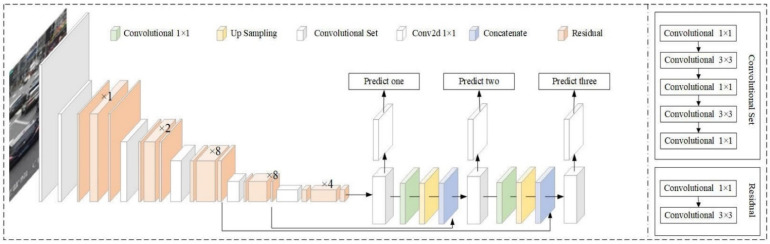
YOLOv3 network architecture that predicts at three scales [64].

YOLO, You Only Look Once, is an object detector that uses features learned by a deep convolutional neural network to detect objects [58]. The architecture of YOLO v3 includes 106 layer fully convolutional layers. It makes predictions of bounding boxes at three scales by downsampling the dimensions of the input image at different layers and extracting features from them. Darknet-53 performs feature extraction that is more powerful and efficient. Up-sampled layers help hold fine features, making it better at detecting small objects. Class predictions for each bounding box are made using cross-entropy loss and logistic regression instead of softmax. The network architecture of the model is shown in Figure 3.

**Figure 4:**
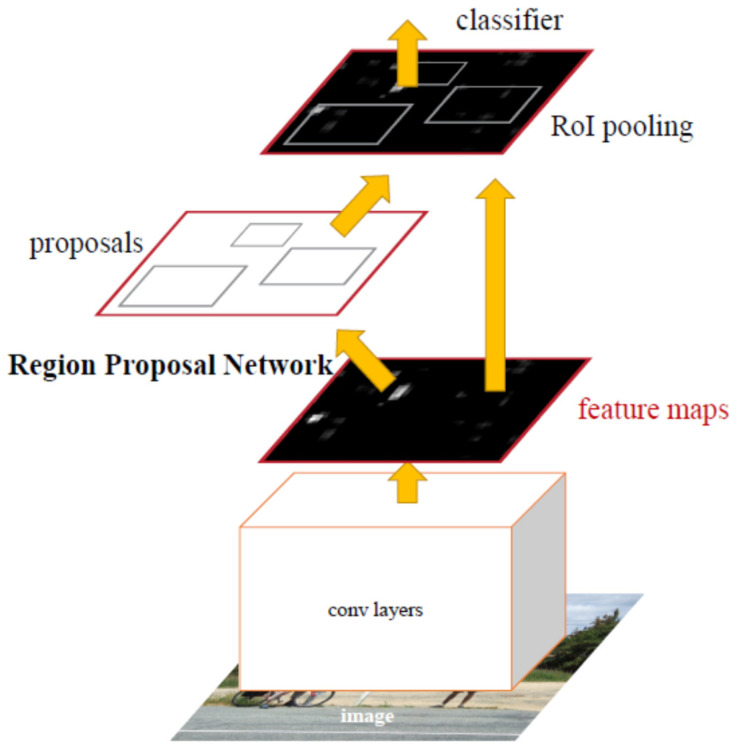
Faster R-CNN system for object detection with RPN [57].

Faster R-CNN uses Region Proposal Networks, a fully convolutional network that simultaneously predicts object bounds and objectness scores, to create potential bounding boxes and afterward runs a classifier on these proposed boxes instead of using Selective Search as a region proposal technique. Classification is followed by a post-processing phase that refines bounding boxes, excluding duplications and score bounding boxes again [65]. Figure 4 summarizes how it proceeds from beginning to end.

Adam optimizer was used with a learning rate of 0.0001. The model was run on Windows OS with NVIDIA GeForce RTX 3080 graphics processor unit. Object detection models give outputs bounding box and class of the objects in input images. The detection performance is evaluated by mean average precision (mAP), recall, and precision metrics. The mAP is also used for Pattern Analysis, Statistical modeling and Computational Learning (PASCAL) Visual Object Classes (VOC) Challenge [66]. It can briefly be described step by step as follows.

Intersection Over Union (IOU) defines how the bounding box is predicted correctly. It is calculated as a ratio of overlap between the predicted bounding box area and the ground truth area. It takes values between 0 and 1, indicating no overlap and exact overlap, respectively, as shown in Equation (1) [6,9,13].

(1) $$\mathrm{IoU}=\frac{\mathrm{area}\left(\mathrm{ground}\;\mathrm{truth}\;\cap^{\;}\mathrm{predicted}\right)}{\mathrm{area}\left(\mathrm{ground}\;\mathrm{truth}\;\cup^{\;}\mathrm{predicted}\right)}$$

Precision refers to how exactly the model identifies relevant objects, while recall measures the model’s ability to propose correct detections among all ground truths, which are given in Equations (2) and (3) [6,9,13]. While comparing two models, a model with high precision and recall value are considered better performance.

(2) $$\mathrm{precision}=\frac{\mathrm{number}\;\mathrm{of}\;\mathrm{correct}\;\mathrm{regions}\;\mathrm{detected}\;}{\mathrm{number}\;\mathrm{of}\;\mathrm{correct}\;\mathrm{regions}\;\mathrm{detected}+\mathrm{number}\;\mathrm{of}\;\mathrm{false}\;\mathrm{regions}\;\mathrm{detected}}$$

(3) $$\mathrm{recall}=\frac{\mathrm{number}\;\mathrm{of}\;\mathrm{correct}\;\mathrm{regions}\;\mathrm{detected}}{\mathrm{number}\;\mathrm{of}\;\mathrm{all}\;\mathrm{regions}}$$

Average Precision represents the area under the precision-recall curve that is evaluated at an IoU threshold. It is defined in Equation (4) [6,9,13].

(4) $$\mathrm{AP}@\mathrm{threshold}=\overset{1}{\underset{0}{\int }}p\left(r\right)\mathrm{dr}$$

The notation of AP@threshold indicates that AP is calculated at a given IoU threshold. For most models, it is considered 0.5 and shown by AP@0.5. AP is calculated for each class in the data, resulting in n-different AP values for n-classes. When these values are averaged, mean Average Precision (mAP) is obtained for n classes with Equation (5) [6,9,13].

(5) $$\mathrm{mAP}@\mathrm{threshold}=\frac{1}{n}\overset{n}{\underset{i=1}{\sum }}{\mathrm{AP}}_{i}$$

Accuracy is a metric used to evaluate classification performance. It refers to the percentage of the correct predictions for the test dataset, as shown in Equation (6). It describes how the model performs for all classes [6,9,13].

(6) $$\mathrm{accuracy}=\frac{\mathrm{number}\;\mathrm{of}\;\mathrm{correct}\;\mathrm{predictions}}{\mathrm{number}\;\mathrm{of}\;\mathrm{all}\;\mathrm{predictions}}$$

Object detection algorithms make predictions with a bounding box and a class label. For each object, the predicted bounding box and ground truth are measured by intersection over union (IoU) [67]. If the IoU value of the prediction is bigger than the IoU threshold, the object is classified as true positive (TP). Precision and Recall are calculated based on the measured IoU and IoU threshold. Average precision (AP) is the area under the Precision-Recall curve. The mean average precision (mAP) is calculated by considering the mean AP over all classes [68].

While mAP@0.5 refers to the mAP when the IoU threshold is 0.5, mAP@0.5–0.95 means the average mAP over different IoU thresholds from 0.5 to 0.95 [69]. Many algorithms, including Faster RCNN, YOLO, use mAP to evaluate the model performance [57,58].

## 3. Results

Findings were categorized into two groups of one-stage and two-stage detectors in structural design. Detection performances of four different architectures were presented based on mAP with thresholds values of 0.5 and 0.5–0.95, which are given in Table 1.

**Figure 5:**
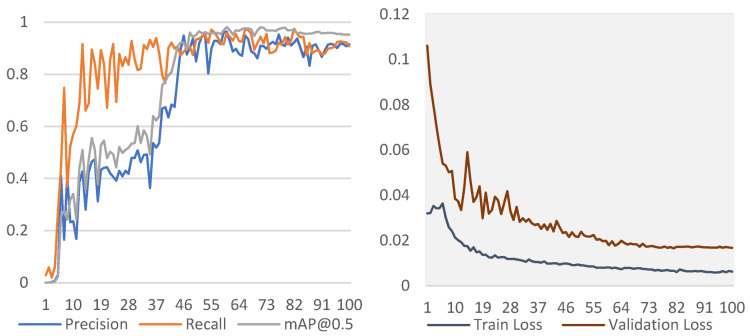
Change of performance metrics and losses for YOLOv3.

As a two-stage technique, Faster RCNN was used as a detector together with three different backbones, ResNet50, AlexNet, and VGG16. They produced mAP@0.5 value of 0.91, 0.86 and 0.87 while mAP@0.5:0.95 value of 0.71, 0.49 and 0.49 respectively. ResNet50 produced the highest mAP performance, while the other two gave a slightly lower rate. On the other hand, YOLOv3 provided the highest rate among all, with a mAP@0.5 value of 0.96 and mAP@0.5:0.95 value of 0.76. The precision and recall were 0.88 and 0.93. Train and validation loss for YOLOv3 were given in Figure 5. Training and validation losses were from a single fold with the best performance.

YOLOv3 outperforms ResNet (*p* = 0.042), AlexNet (*p* = 0.011) and VGG16 (*p* = 0.015). It was not seen that there was a significant difference between ResNet and AlexNet (*p* = 0.158)–VGG16 (*p* = 0.193). Accuracy is calculated for each model and used for statistical analysis. It was presented in Table 2.

YOLOv3 performed better than AlexNet (*p* = 0.016) and VGG16 (*p* = 0.001) in classification accuracy, but there was not a statistically significant difference between the classification accuracies of YOLOv3 and ResNet (*p* = 0.079). Among backbones used in Faster RCNN, a statistically significant difference was not seen between ResNet and AlexNet (*p* = 0.085)–VGG16 (*p* = 0.068).

**Figure 6:**
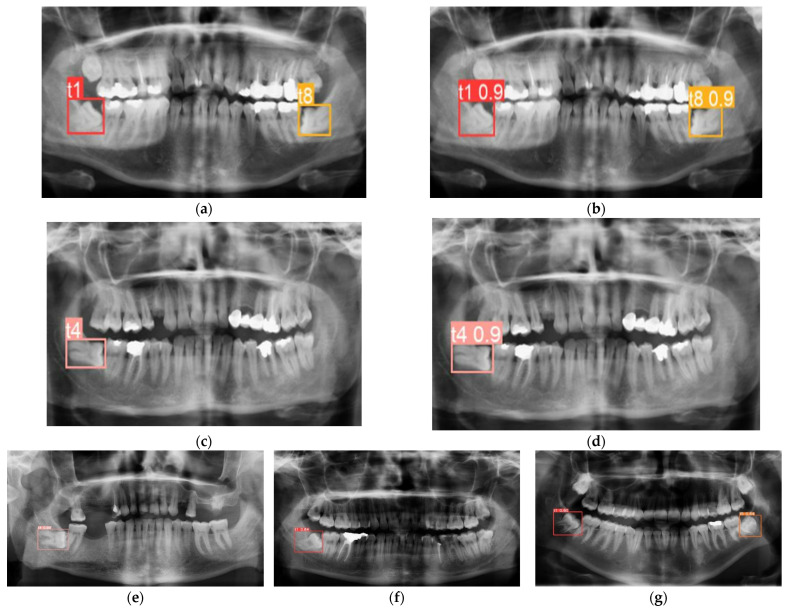
Detection result samples from YOLOv3 (**a**–**d**), ground truths (**a**–**c**), predictions (**b**–**d**) and Faster RCNN with ResNet (**e**), AlexNet (**f**) and VGG16 (**g**).

Findings were also shown on panoramic radiographs with annotations and predictions together. Figure 6 demonstrates how accurately they were detected. Figure 6a–c showed ground truth annotations for the impacted teeth, b-d showed corresponding predictions from the proposed solution using YOLOv3. They were detected and assigned to the proper classes. Figure 6e–g demonstrates random detection result samples from ResNet, AlexNet, and VGG16.

The detection performance for each class was also investigated. It was seen that t1, t4, t5 and t8 showed the mAP@0.5 rate of 0.96, 0.98, 0.984, 0.995 and AP@0.5:0.95 rate of 0.774, 0.775, 0.793, 0.791 respectively. Table 3 shows the performance metrics of each class for the solution with YOLOv3.

mAP at IOU equals to 0.5 are widely accepted detection metric for many real-life detection applications [13,57,58,66]. YOLOv3 presented promising performance with the highest accuracy, indicating that YOLOv3 was a successful detector at detecting third-molar impacted teeth. It was superior to the Faster-RCNN and its use with ResNet50, AlexNet, and VGG16 in terms of mAP and classification accuracy.

## 4. Discussion

A significant increase in the number of studies on artificial intelligence-based decision support systems has been seen in the field of dentistry as well as other fields of healthcare. In dentistry, an automated analysis and interpretation system in which radiographs are automatically analyzed to find defects is a fundamental goal. Previous studies used images from different imaging modalities like panoramic, periapical, and bitewing radiographs for detection, segmentation, and classification purposes. Common topics included studies on tooth detection, tooth numbering, and many other conditions like carries, lesions, anatomical structures, cysts, etc. [17,18,19,20,21,22,23,24,25,26,27,46,47,48,49,50,51,52,53,54,70]. Contrarily, it was observed that previous studies on mandibular third molar detection alone were rare and explored only one model architecture performance.

Lee et al. (2018) suggested a deep learning model to diagnose and predict periodontally compromised teeth. It consisted of 1740 periapical radiographic images, resulting in diagnostic accuracy of 76.7% for molar teeth. It was concluded that the CNN algorithm was helpful for diagnosing periodontally compromised teeth with different expectations of improved systems for better performance in time [71]. They compared the agreement between the expert observer and AI application. The proposed work presented a higher performance in accuracy for YOLOv3 and Faster RCNN ResNet50. Other metrics were not reported.

Faure et al. (2021) proposed a method to automatically diagnose impacted teeth using Faster-RCNN with ResNet101, identifying impacted teeth with performances between 51.7% and 88.9% [38]. Angulation was not investigated; only one class for impacted third molars was used. The proposed work provided a more elaborative analysis by using two detectors with three backbones and a higher number of classes.

Basaran et al. (2021) suggested a model for the diagnostic charting of ten dental conditions, including impacted teeth, in panoramic radiography. Their model was based on Faster R-CNN Inception v2, including 1084 graphs with 796 impacted teeth. It was reported that sensitivity and precision for impacted teeth were 0.96 and 0.77 [41]. When it was compared, this work presented higher precision, but mAP was not reported.

Tobel et al. (2017) used CNNs to develop an automated technique to monitor the development stages of the lower third molar on panoramic radiographs. They classified their growth into ten classes. They concluded that the performance was similar to staging by human observers but needed to be optimized for age estimation [72].

Vinayahalingam et al. (2019) implemented CNNs to detect and segment inferior alveolar nerve and lower third molars on panoramic radiographs. The mean dice-coefficient for the third molar was 0.947 ± 0.033 [26]. Contrary to traditional simple architectures, deep CNNs succeed in edge detection thanks to multiple convolutional and hidden layers featuring hierarchical feature presentation [73].

Kuwada et al. (2020) performed detection and classification for impacted supernumerary teeth in the anterior maxillary area [4]. This region was completely different from the region of third molars. Zhang et al. (2018) used 15 patient-related factors to predict postoperative facial swelling following impacted mandibular third molars extraction [39]. They used angulation of the third molar with respect to the second molar as a parameter but did not perform detection. Orhan et al. (2021) performed segmentation to detect third molar teeth with a precision value of 0.77 [40]. They used Cone-Beam Computed Tomography images and compared agreement between the human observer and AI application. The proposed work used panoramic images with higher precision in addition to evaluation metrics for detection.

Considering previous works, this work demonstrated an immediate and comprehensive solution for automated detection of mandibular third molar teeth using two types of detection techniques for the first time. This work also included two different third molar impaction classes. Previous works had only one class for all third molars, which limited the corresponding comparison. Moreover, many previous works performed classification and segmentation tasks for different purposes. This work focused only mandibular third molar detection problem. Although mAP was a standard evaluation metric for detection tasks in the computer vision field, it was not reported in previous works. Briefly, it was not always possible to directly compare each work because of incompatibilities between (i) type of radiography used, (ii) evaluation metrics used, and (iii) purposes. 

Multi-label classification used in YOLOv3 performed better for datasets with overlapping labels than using softmax, which assumed each bounding box had only one class, which was not the case in real-life applications. Additional techniques such as advanced image pre-processing, data augmentation, and more data can improve the proposed solution’s results and make it more robust. 

This work has two limitations. First, the amount of data is limited. More panoramic radiographs will be collected and annotated to perform deep learning for more robust, reliable results. Second, mandibular third molars were used in this work due to their wider prevalence. Later, maxillary third molars will be analyzed as more data are collected.

## 5. Conclusions

The proposed solution aims to help dentists in their decision-making process. It is shown that four different models are successful in detecting third molars. The use of machine learning in dentistry has significant potential in diagnosis with high accuracy and precision. Diagnostic tools based on state-of-the-art deep learning models are reliable and robust auxiliary techniques for clinical decision-making, resulting in more efficient treatment planning for patients and clinician health management. In time, AI-based devices can be used as a standard tool in clinical practice and play a crucial role in providing diagnostic recommendations.

## Figures and Tables

**Table 1:** Detection performances of two detectors, one with three different backbones.

	Fold	mAP@0.5	mAP@0.5:0.95
One-stage technique			
YOLOv3	1	0.941	0.751
	2	0.979	0.783
	3	0.936	0.746
	4	0.981	0.761
	5	0.98	0.771
	Avg	0.96	0.76
Two-stage technique			
Faster RCNN–ResNet50	1	0.912	0.628
	2	0.904	0.673
	3	0.86	0.646
	4	0.944	0.71
	5	0.953	0.713
	Avg	0.91	0.71
Faster RCNN–AlexNet	1	0.814	0.433
	2	0.878	0.518
	3	0.773	0.47
	4	0.916	0.52
	5	0.923	0.513
	Avg	0.86	0.49
Faster RCNN–VGG16	1	0.838	0.464
	2	0.89	0.486
	3	0.802	0.423
	4	0.898	0.484
	5	0.937	0.583
	Avg	0.87	0.49

**Table 2:** Classification accuracies.

Fold	YOLOv3	Faster RCNN–ResNet50	Faster RCNN–AlexNet	Faster RCNN–VGG16
1	0.824	0.814	0.636	0.674
2	0.86	0.727	0.68	0.693
3	0.834	0.713	0.529	0.653
4	0.897	0.856	0.76	0.736
5	0.891	0.854	0.81	0.792
Avg	0.86	0.79	0.68	0.7

**Table 3:** Inter-class detection performances for the solution with YOLOv3.

Class	AP@0.5	AP@0.5:0.95	Precision	Recall
t1—mesioangular left	0.96	0.774	0.849	0.95
t4—horizontal left	0.98	0.775	0.96	0.833
t5—mesioangular right	0.984	0.793	0.908	0.987
t8—horizontal right	0.995	0.791	0.88	1

## Data Availability

The data supporting this study’s findings are not available and accessible due to ethical issues, patients’ and institutions’ data protection policies.
